# Overview of clinical status, treatment, and long-term outcomes of pediatric autosomal-dominant polycystic kidney disease: a nationwide survey in Taiwan

**DOI:** 10.1038/s41598-024-67250-z

**Published:** 2024-07-15

**Authors:** Chia-Yi Chin, Wan-Ting Huang, Jen-Hung Wang, Je-Wen Liou, Hao-Jen Hsu, Ming-Chun Chen

**Affiliations:** 1 Department of Pediatrics, Hualien Tzu Chi Hospital, Buddhist Tzu Chi Medical Foundation, Hualien, 97004 Taiwan; 2 School of Medicine, Tzu Chi University, Hualien, 97004 Taiwan; 3 Epidemiology and Biostatistics Center, Hualien Tzu Chi Hospital, Buddhist Tzu Chi Medical Foundation, Hualien, 97004 Taiwan; 4 Department of Biochemistry, School of Medicine, Tzu Chi University, Hualien, 97004 Taiwan; 5 Department of Biomedical Sciences and Engineering, Tzu Chi University, Hualien, 97004 Taiwan

**Keywords:** Autosomal-dominant polycystic kidney disease, Pediatric, Complication, Medication, Population-based cohort study, Nephrology, Cardiology

## Abstract

This retrospective study investigated the incidence, medication use, and outcomes in pediatric autosomal-dominant polycystic kidney disease (ADPKD) using Taiwan's National Health Insurance Research Database (NHIRD). A 1:4 matched control group of individuals included in the NHIRD during the same period was used for comparative analyses. A total of 621 pediatric patients were identified from 2009 to 2019 (mean age, 9.51 ± 6.43 years), and ADPKD incidence ranged from 2.32 to 4.45 per 100,000 individuals (cumulative incidence, 1.26–1.57%). The incidence of newly developed hypertension, anti-hypertensive agent use, nephrolithiasis, and proteinuria were significantly higher in the ADPKD group than the non-ADPKD group (0.7 vs. 0.04, 2.26 vs. 0.30, 0.4 vs. 0.02, and 0.73 vs. 0.05 per 100 person-years, respectively). The adjusted hazard ratios for developing hypertension, proteinuria, nephrolithiasis and anti-hypertensive agent use in cases of newly-diagnosed pediatric ADPKD were 12.36 (95% CI 4.92–31.0), 13.49 (95% CI 5.23–34.79), 13.17 (95% CI 2.48–69.98), and 6.38 (95% CI 4.12–9.89), respectively. The incidence of congenital cardiac defects, hematuria, urinary tract infections, gastrointestinal diverticulosis, dyslipidemia, and hyperuricemia were also higher in the ADPKD group. Our study offers valuable insights into the epidemiology of pediatric ADPKD in Taiwan and could help in formulating guidelines for its appropriate management.

## Introduction

Autosomal-dominant polycystic kidney disease (ADPKD), a ciliopathy characterized by diffuse cyst formation, is the most prevalent hereditary renal disease, with an estimated prevalence ranging from 1 in 500 to 2,500 individuals worldwide^1,2^. ADPKD accounts for 5% of all cases of end-stage renal disease and 10% of cases in individuals under 65 years of age^3^. Loss of function mutations in PKD1 (chromosome 16p13.3) and PKD2 (chromosome 4q21)—which encode for polycystin-1 (PC1) and polycystin-2 (PC2), respectively—are responsible for approximately 95% of all ADPKD cases^3,4^. These mutations disrupt the molecular pathways that control cellular proliferation, tubulogenesis, and fluid secretion, eventually leading to the development of fluid-filled cysts^4^.

A high incidence of cardiovascular, renal, and gastrointestinal complications has been reported among patients with ADPKD^5^, including children and adolescents^6^. Approximately 3% of children with ADPKD-causing mutations develop progressive structural kidney disease at an unusually early age or at an accelerated rate, with the disease even manifesting in utero in some cases^7^. Thus, early childhood interventions to mitigate the progression of ADPKD to chronic renal failure could significantly impact individual and public health and medical resource requirements in the long term^6^.

The incidence of ADPKD and associated complications vary geographically, ethnically, and methodologically^1,2,6^. However, there are no reports on the epidemiology and current characteristics of the pediatric ADPKD population in Taiwan. Moreover, there is a lack of long-term follow-up data on medication use, clinical outcomes, comorbidities, and complications, and most previously published reports relied on small, single-center cohorts.

The National Health Insurance Research Database (NHIRD) provides access to 99.8% of healthcare data in Taiwan, facilitating population-based epidemiological research and evaluation of disease incidence and healthcare burdens^8^. This study utilized NHIRD data to investigate nation-wide trends in pediatric ADPKD incidence, medication use, and disease outcomes and complications in Taiwan.

## Methods

### Setting and data sources

Over 90% of hospitals and clinics in Taiwan are included under the government-sponsored Taiwan National Health Insurance program. Our population-based retrospective study used the Health and Welfare Data Science Center (HWDC) datasets extracted from the Taiwan NHIRD, which contains healthcare details and vital status data of 99.8% of Taiwan’s 23 million residents (https://nhird.nhri.org.tw/en/index.html)^8,9^, to identify patients with incident ADPKD among all individuals registered during the period spanning January 2009 to December 2019, as disclosed by the Collaboration Center of Health Information Application of Taiwan. The diagnosis codes were based on the 9^th^ and 10^th^ revisions of the International Classification of Diseases, Clinical Modification codes (ICD-9-CM for 2009 to 2015 and ICD-10-CM 2016 onwards). Incidence rates were calculated using public national birth data from the Taiwanese Ministry of the Interior as the denominator.

### Ethics approval and patient selection

The study was conducted in accordance with the guidelines of the Declaration of Helsinki and approved by the Ethics Committee of the Protection of Human Subjects Institutional Review Board of Tzu Chi Hospital (approval no: IRB111-023-B, approved on April 21, 2022); the requirement for informed consent was waived due to the retrospective nature of the study. The ADPKD cohort comprised patients with at least one hospitalization or two outpatient visit records for ADPKD (ICD-9-CM codes: 753.12, 753.13; ICD-10 codes: Q61.2, Q61.3). The first date of diagnosis was used as the index date^9^. Patients with invalid or missing personal data were excluded from the analysis. Subjects with comorbidities reported before the index date were included in baseline disease analysis but excluded from further ADPKD-related complication analyses in this study^8^. Control subjects, matched 1:4 to the ADPKD group by sex, age, and index date (between 2009 and 2019), were selected from the remaining NHIRD cohort and using the same exclusion criteria.

### Evaluation of ADPKD-related complications

Several cardiovascular, renal, and gastrointestinal complications are associated with of ADPKD^3,5^. We analyzed ADPKD-related complications for patients and controls who had at least one hospitalization or two outpatient visits, identified by the relevant ICD-9 or ICD-10 codes, with a minimum interval of 30 days from the index date, as detailed in Table XXX2. In assessing the clinical outcomes of children with very-early onset (VEO) ADPKD, diagnosed within the first 1.5 years of life, we conducted a subgroup analysis focusing on ADPKD patients younger than 1.5 years, as presented in Table XXX4.

### Evaluation of ADPKD-related medication use

To analyze the use of anti-hypertensive medications in newly diagnosed ADPKD patients, the Anatomical Therapeutic Chemical Classification (ATC) system was used to analyze medication use. Previously reported medications for symptomatic ADPKD control, including statins (ATC codes: C10AA, C10BX03) and anti-hypertensive agents (ATC codes: C02–C04, C07–C09) were assessed^5,6^. Common anti-hypertensive agents used for blood pressure control were grouped into six categories: (1) angiotensin-converting enzyme inhibitors (ACEis) and angiotensin receptor blockers (ARBs), (2) beta blockers, (3) calcium channel blockers (CCBs), (4) diuretics, (5) aldosterone blockers, and (6) others^10,11^. Individuals who had used above medications for more than 30 days in the year prior to the index date were excluded. Therefore, the medications we refer to are those newly initiated after the diagnosis of ADPKD. Only medications used > 30 days after the index date were included in the analysis.

### Statistical analysis

Annual incidence rates based on age and sex were calculated by dividing the number of new cases in the ADPKD and non-ADPKD groups based on the corresponding age- and sex-specific population data for the study period obtained from the Taiwanese Ministry of the Interior. Descriptive statistics data are presented in terms of absolute numbers and percentages. Comparison of two incidences was performed using the Chi2-statistic. Univariable and multivariable Cox proportional hazards models were used to estimate the risk of different complication for two groups. SAS version 9.4 (SAS Institute Inc., Cary, NC, USA) was used for all statistical analyses. Statistical significance was set at *P* < 0.05.

## Results

### Demographic information of pediatric patients with ADPKD

**Figure 1:**
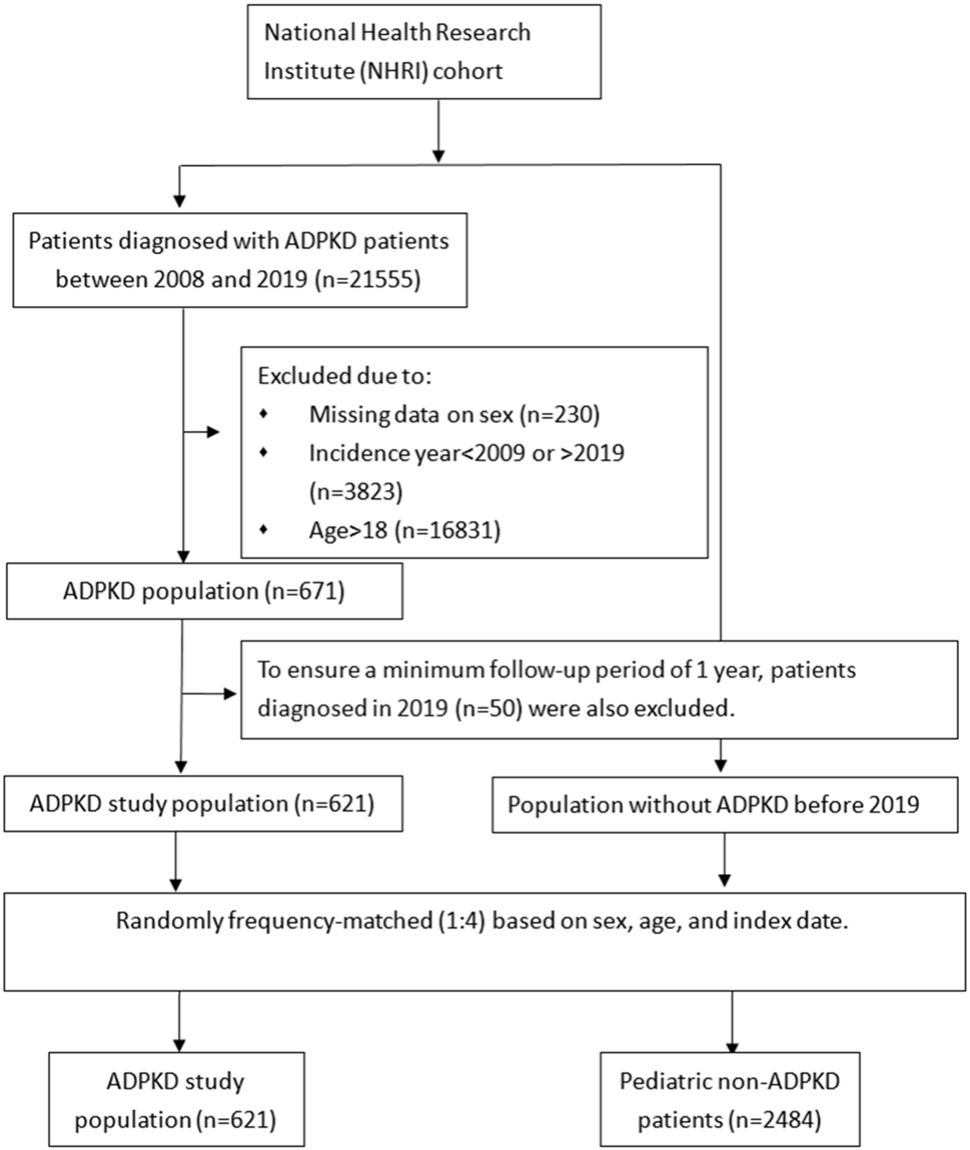
Flowchart of patient selection for the pediatric ADPKD group and the matched non-ADPKD group. ADPKD, autosomal-dominant polycystic kidney disease.

A total of 21,555 patients were diagnosed with ADPKD (Fig. 1) between 2008 and 2019 in Taiwan according to the National Health Research Institute (NHRI) data. Patients over 18 years of age (n = 16,831), with missing information regarding sex (n = 230), and first diagnosed before 2009 (n = 3823) were excluded. To ensure a minimum follow-up period of 1 year for the study population, children diagnosed with ADPKD in 2019 (n = 50) were excluded from the outcome analysis.

Finally, this study included and analyzed 621 pediatric patients newly diagnosed with ADPKD between January 1, 2009 and December 31, 2018 and 2484 controls. Patients were followed-up from the index date until the occurrence of an event, death, or the end of the study on December 31, 2019.

**Figure 2:**
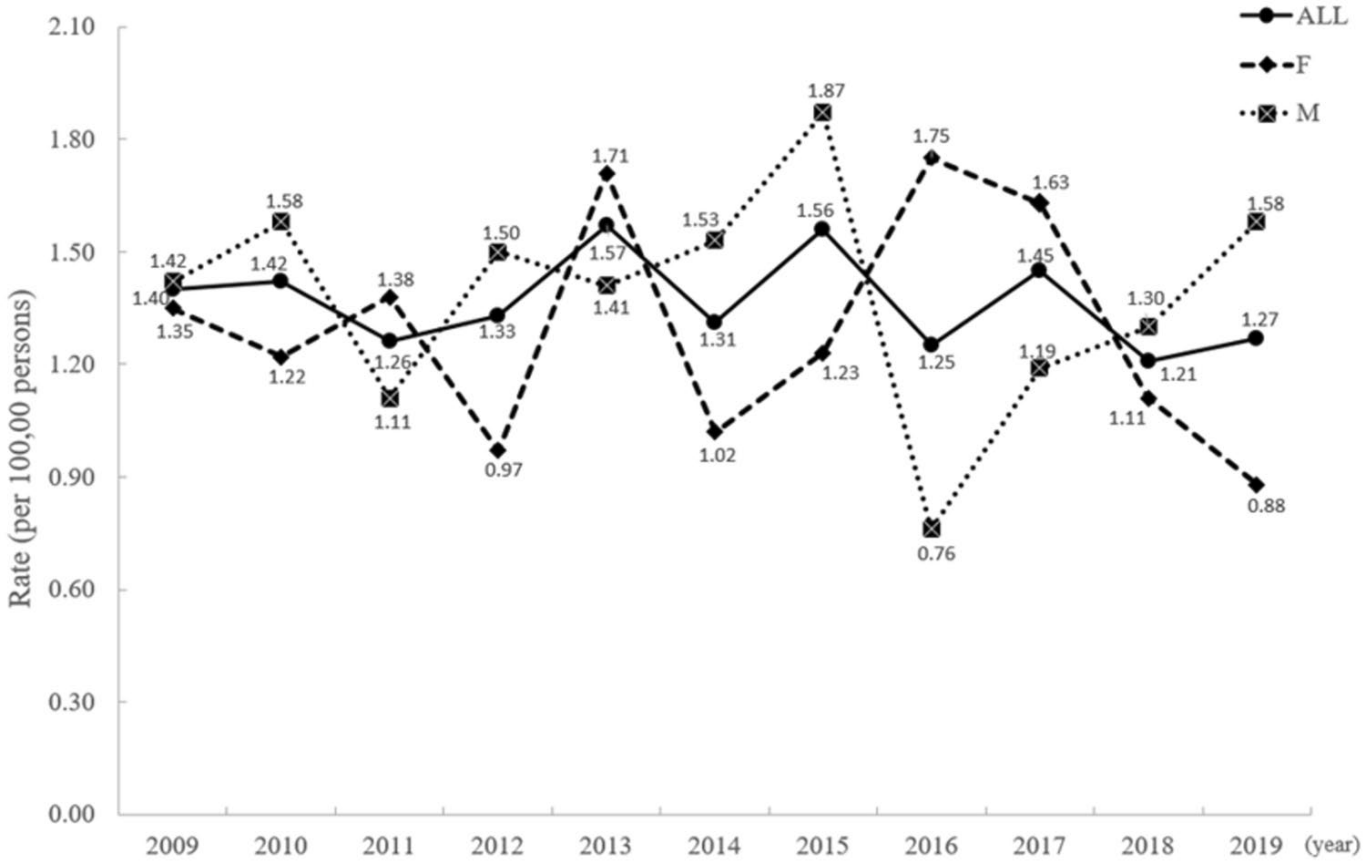
Incidence rates in the ADPKD population by gender from 2009 to 2019.

The prevalence of ADPKD in the pediatric population between 2008 and 2019 was 2.32 to 4.45 per 100,000 persons. The cumulative incidence rate of pediatric ADPKD from 2009 to 2019 was 1.26–1.57% (Fig. 2).

The characteristics and common comorbidities of patients with ADPKD in our cohort are shown in Table 1. The mean age was 9.51 ± 6.43 years, and male sex was more common (53.8%). Ten percent of patients had urinary tract infections before being diagnosed with ADPKD, and 3.87% and 2.24% children newly diagnosed with ADPKD already had hematuria and proteinuria/albuminuria, respectively. Chronic kidney disease (CKD) was already diagnosed in 1.2% of children with ADPKD. Approximately 2% of patients had hypertension and congenital heart defects and nearly 5% had already used anti-hypertensive drugs before being diagnosed with ADPKD.

**Table 1:** Descriptive results of new-onset ADPKD population in children aged 18 and below from 2009 to 2019.

Variable	n	%
AGE (Mean , SD)	9.51	6.43
Gender		
Boy	361	53.80
Girl	310	46.20
Baseline disease		
CV system		
Hypertension	16	2.38
Congenital cardiac defects	17	2.53
Mitral valve prolapse	3	0.45
Renal system		
Proteinuria/Albuminuria	15	2.24
Hematuria	26	3.87
Urinary tract or cyst infections	72	10.73
Chronic kidney disease	8	1.19
Gastrointestinal system		
Colonic diverticulum /intestinal diverticulum	3	0.45
Inguinal hernias	5	0.75
Other		
Dyslipidemia	5	0.75
Baseline drug use		
Antihypertensive drug	32	4.77

### Complications in pediatric ADPKD

**Figure 3:**
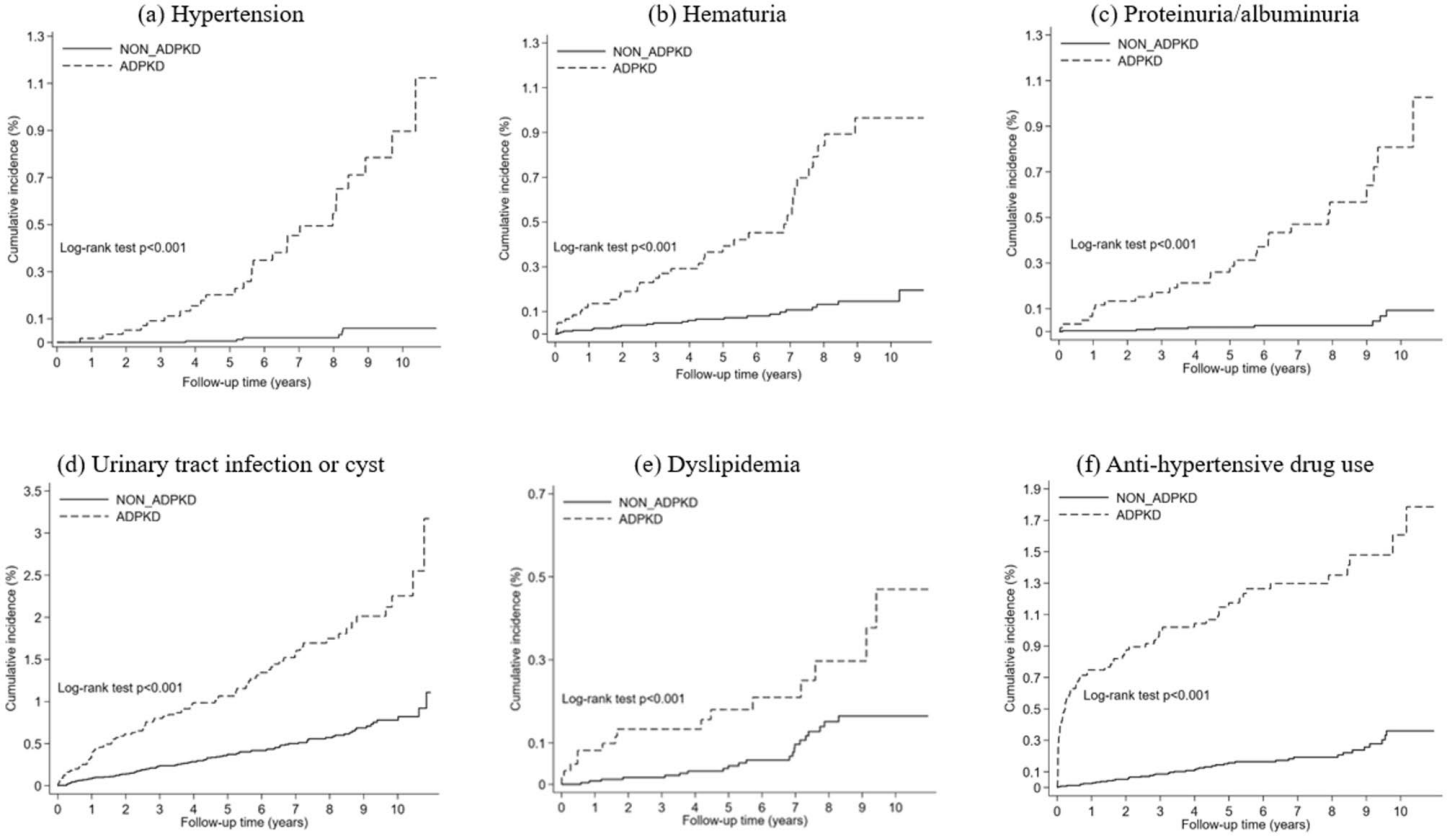
Kaplan–Meier curves for the assessed events in the ADPKD and non-ADPKD groups.

Details regarding the patterns of various complications and the corresponding characteristics of pediatric patients with ADPKD in Taiwan are presented in Tables 2 and 3 and Fig. 3.

**Table 2:** The incidence rate of diseases among patients with ADPKD study population and without ADPKD.

Outcome	ADPKD				Non ADPKD				IRR	*P* value
	n	Event	py	IR*	n	Event	py	IR *		
CV system										
Hypertension	606	26	3640	0.7	2424	6	14,967	0.04	17.82	** < 0.001**
Congenital cardiac defects	606	12	3698	0.3	2424	26	15,029	0.17	1.88	0.067
Mitral valve prolapse	619	6	3785	0.2	2476	13	15,320	0.08	1.87	0.198
Renal system										
Proteinuria/albuminuria	608	27	3699	0.73	2432	8	15,234	0.05	13.90	** < 0.001**
Hematuria	595	33	3554	0.93	2380	23	14,690	0.16	5.93	** < 0.001**
Urinary tract or cyst infections	553	80	3153	2.54	2212	105	13,394	0.78	3.24	**0.006**
Nephrolithiasis	619	15	3760	0.40	2476	3	153,846	0.02	20.46	** < 0.001**
Chronic kidney disease	613	23	3694	0.62	2452	0	-	-	-	-
Gastrointestinal system										
Diverticulosis	618	6	3769	0.2	2472	5	15,338	0.03	4.88	** < 0.001**
Inguinal hernias	616	5	3758	0.13	2464	16	15,213	0.11	1.27	0.645
Other										
Dyslipidemia	616	15	3729	0.4	2464	21	15,303	0.14	2.93	** < 0.001**
Hyperuricemia	620	7	3791	0.2	2480	4	15,410	0.03	7.11	** < 0.001**
Drug use										
Antihypertensive drug	591	73	3234	2,26	2364	43	14,377	0.30	7.55	** < 0.001**
Statins	619	8	3774	0.21	2476	3	153,846	0.02	10.87	** < 0.001**

Significant values are in bold.

*ADPKD* autosomal dominant polycystic kidney disease, *IR* incidence rate, *IRR* incidence rate ratio, *py* person follow year.

*IR per 100 person-year.

**Table 3:** Estimating the crude hazard ratio (HR) and adjusted hazard ratio (aHR) of disease among patients with ADPKD study population.

Outcome	Crude HR (95% CI)	*P* value	Adjust HR (95% CI)	*P* value
CV system				
Hypertension	17.19 (7.08, 41.77)	** < 0.001**	12.36 (4.92, 31)	** < 0.001**
Congenital cardiac defects	1.97 (0.98, 3.93)	0.056	2.34 (1.11, 4.9)	**0.026**
Mitral valve prolapse	1.85 (0.70, 4.86)	0.214	1.82 (0.63, 5.23)	0.268
Renal system				
Proteinuria/albuminuria	13.5 (6.13, 29.71)	** < 0.001**	13.49 (5.23, 34.79)	** < 0.001**
Hematuria	5.94 (3.46 , 10.19)	** < 0.001**	5.55 (3.07, 10.00)	** < 0.001**
Urinary tract or cyst infections	3.53 (2.60 , 4.79)	** < 0.001**	3.50 (2.53, 4.85)	** < 0.001**
Nephrolithiasis	19.71 (5.7, 68.11)	** < 0.001**	13.17 (2.48, 69.98)	**0.003**
Gastrointestinal system				
Diverticulosis	3.33 (1.02, 10.92)	**0.047**	3.89 (1.12, 13.44)	**0.032**
Inguinal hernias	1.23 (0.45, 3.36)	0.684	0.73 (0.16, 3.28)	0.679
Other				
Dyslipidemia	2.83 (1.46, 5.50)	**0.002**	3.66 (1.77, 7.59)	**0.001**
Hyperuricemia	7.00 (2.05, 23.91)	**0.002**	8.00 (2.00, 31.99)	**0.003**
Drug use				
Antihypertensive drug	8.01 (5.37, 11.94)	** < 0.001**	6.38 (4.12, 9.89)	** < 0.001**
Statins	10.67 (2.83, 40.2)	** < 0.001**	8.66 (1.64, 45.64)	**0.011**

Significant values are in bold.

*Adjusted analyses included the baseline variables: age, sex, hypertension, congenital cardiac defects, Mitral valve prolapse, urinary tract or cyst infections , prolapse , proteinuria/albuminuria, hematuria, chronic kidney disease, colonic diverticulum/ intestinal diverticulum, Inguinal hernias, dyslipidemia, antihypertensive drug.

#### Cardiovascular system

The incidence of newly developed hypertension was significantly higher among children with ADPKD from 2009 to 2018 (Table 2) than that among children without ADPKD (0.7 versus 0.04 per 100 person-years, *P* < 0.001; incidence rate ratio [IRR], approximately 17.8). The incidences of congenital cardiac defects (0.3 versus 0.17 per 100 person-years, *P* = 0.067) and mitral valve prolapse (0.2 versus 0.08 per 100 person-years, *P* = 0.198) were higher among children with ADPKD. However, these differences did not reach statistical significance.

The cumulative incidence of hypertension (Fig. 3a) was significantly higher among children with ADPKD than in those without ADPKD (log-rank test, *P* < 0.001). The hazard ratio (HR) of developing hypertension (Table 3) was 17.19 (95% confidence interval [CI]: 7.08 to 41.77) and the adjusted HR (aHR) was 12.36 (95% CI: 4.92 to 31.0) in children newly diagnosed with ADPKD. The HR and aHR of newly diagnosed congenital cardiac defect were 1.97 (95% CI 0.98–3.93) and 2.34 (95% CI 1.11–4.90), respectively. The HR for newly diagnosed mitral valve prolapse was not statistically significant (Table 3). In children aged 1.5 years and younger, as documented in Table 4, the incidence of hypertension was 0.39 per 100 person-year. The incidence of congenital cardiac defects was higher among children with ADPKD (0.81 vs. 0.42 per 100 person-years, *P* = 0.179), although this difference did not achieve statistical significance.

**Table 4:** The incidence rate of diseases among patients aged 1.5 years and younger in the ADPKD study population and the non-ADPKD population.

Outcome	ADPKD		Non ADPKD		IRR	*P* value
	n	IR	n	IR		
CV system						
Hypertension	130	0.39	520	0	–	–
Congenital cardiac defects	125	0.81	500	0.42	1.92	0.179
Mitral valve prolapse	131	0	524	0	–	–
Renal system						
Proteinuria/Albuminuria	129	0.26	516	0	–	–
Hematuria	131	0.65	524	0.12	5.18	**0.006**
Urinary tract or cyst infections	106	5.22	424	1.67	3.12	** < 0.001**
Nephrolithiasis	131	0	524	0	–	–
Chronic kidney disease	131	0.52	524	0	–	–
Gastrointestinal system						
Diverticulosis	130	0.26	520	0	–	–
Inguinal hernias	128	0.40	512	0.39	1.02	0.974
Other						
Dyslipidemia	131	0	524	0	–	–
Hyperuricemia	131	0	524	0.03	–	–
Drug use						
Antihypertensive drug	129	1.52	516	0.13	12.00	** < 0.001**
Statins	131	0	524	0	–	–

Significant values are in bold.

#### Renal system

The incidence of nephrolithiasis among children with ADPKD was 0.4 per 100 person-years, which was significantly higher than that among children without ADPKD in (0.02 per 100 person-years, *P* < 0.001; IRR, 20.5). The incidence of proteinuria/albuminuria and hematuria were also significantly higher in children with ADPKD compared to those without ADPKD (0.73 versus 0.05 per 100 person-years, *P* < 0.001; IRR, 13.9 for proteinuria/albuminuria and 0.93 versus 0.16 per 100 person-years, *P* < 0.001; IRR: 5.9 for hematuria).

The cumulative incidence of proteinuria or albuminuria, hematuria, and urinary tract or cystic infection were significantly higher in children with ADPKD (Fig. 3b–d). The HR for developing renal system disorders among children newly diagnosed with ADPKD are shown in Table 3. The HR and aHR for developing proteinuria or albuminuria were 13.50 (95% CI 6.13–29.71) and 13.49 (95% CI 5.23–34.79), respectively. The HR and aHR for developing nephrolithiasis during follow-up were 19.71 (95% CI 5.70–68.11) and 13.17 (95% CI 2.48–69.98), respectively. The risk of hematuria and urinary tract or cystic infection were also significantly higher among children with ADPKD. There were 23 CKD events in the ADPKD group and the incidence ratio was 0.62 per 100 person-years. No CKD event was documented in children without ADPKD. In children aged 1.5 years and younger, as outlined in Table 4, the incidence rates for proteinuria/albuminuria and CKD were 0.26 and 0.52 per 100 person-year, respectively. The incidence of hematuria and urinary tract or cystic infection was also significantly elevated in children with ADPKD, with rates of 0.65 compared to 0.12 per 100 person-years (*P* = 0.006; IRR: 5.18 for hematuria) and 5.22 versus 1.67 per 100 person-years (*P* < 0.001; IRR: 3.12 for urinary tract or cystic infection).

#### Other systems

Specific diseases associated with ADPKD in adults, specifically diverticulum in the colon or intestine, , dyslipidemia, and hyperuricemia, was significantly higher among children with ADPKD than among children without ADPKD (0.2 versus 0.03, 0.4 versus 0.14, and 0.2 versus 0.03 per 100 person-years, all *P* < 0.001).

ADPKD children had higher inguinal hernia rates, but not significantly different from non-ADPKD (0.13 vs 0.11, *P* = 0.645).

The cumulative incidence of dyslipidemia (Fig. 3e) was significantly higher in children with ADPKD than in those without ADPKD (log-rank test, *P* < 0.001). The HR and aHR of developing dyslipidemia (Table 3) were 2.83 (95% CI 1.46–5.50) and 3.66 (95% CI 1.77–7.59) and those for hyperuricemia were 7.0 (95% CI 2.05–23.91) and 8.0 (95% CI 2.0–31.99), respectively. The HR and aHR of developing diverticulum in the colon or intestine during follow-up were 3.33 (95% CI 1.02–10.92) and 3.89 (95% CI 1.12–13.44). The risk of developing inguinal hernia was not statistically significant.

### Medication use

**Figure 4:**
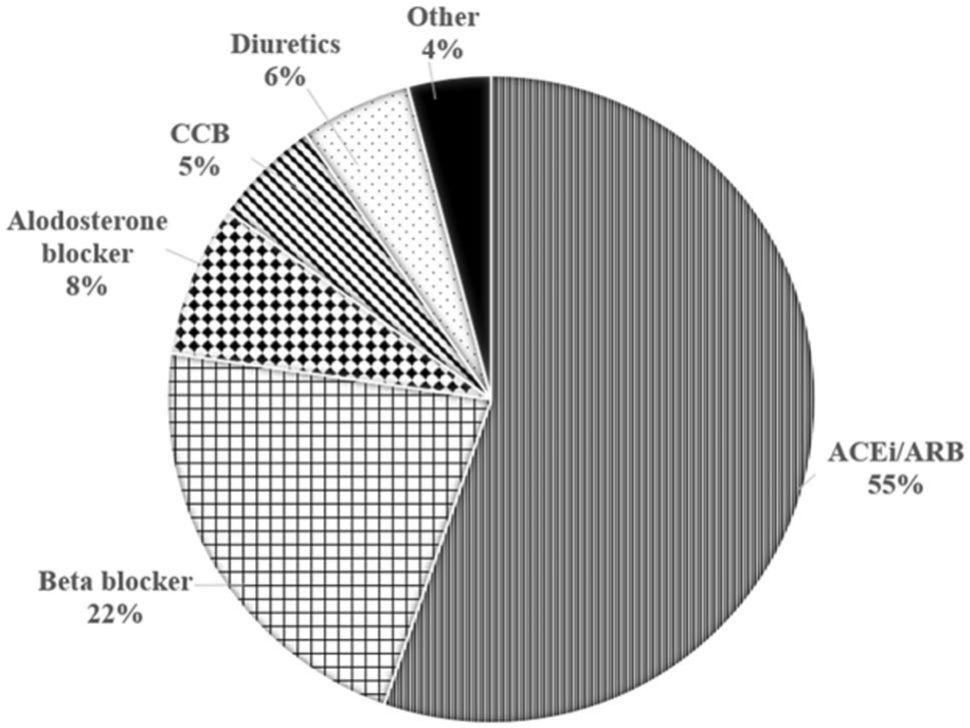
Distribution of anti-hypertensive medications used in the ADPKD population. ACEi, angiotensin-converting enzyme inhibitors; ARB, angiotensin receptor blockers.

The incidence of anti-hypertensive agent (2.26 vs 0.30 per 100 person-years, *P* < 0.001) and statin (0.21 vs 0.02 per 100 person-years, *P* < 0.001) use in ADPKD children (Table 2) were significantly higher than those among non-ADPKD children (IRR for anti-hypertensive agent and statin use, 7.55 and 10.87, respectively). A higher incidence of anti-hypertensive agent use was also found in age 1.5 years or younger (1.52 versus 0.13 per 100 person-years, *P* < 0.001) (Table 4). The cumulative incidence of anti-hypertensive medication use (Fig. 3f) was significantly higher in children with ADPKD than in those without ADPKD (log-rank test, *P* < 0.001). The HR and aHR for using anti-hypertensive medications among children newly diagnosed with ADPKD (Table 3) were 8.01 (95% CI 5.37–11.94) and 6.38 (95% CI 4.12–9.89). Furthermore, the risk of statin use was significantly higher among children with ADPKD (HR: 10.67 [95% CI 2.83–40.20]. aHR: 8.66 [95% CI 1.64–45.64]). With regard to the anti-hypertensive medication categories, ACEis and ARBs accounted for 55%, beta-blockers for 22%, aldosterone antagonists for 8%, diuretics for 6%, calcium channel blockers for 5%, and others for 4%, respectively (Fig. 4).

## Discussion

This nationwide retrospective cohort study used the NHRI database to assess the incidence, comorbidities, and medication patterns of ADPKD in the pediatric population in Taiwan. In our cohort, the prevalence of pediatric ADPKD was 2.32–4.45 per 100,000 persons, which was lower than the ADPKD prevalence among adults (9.3–47.6 per 100,000 persons)^1,2,12^; the cumulative incidence rate was 1.26% to 1.57%. The prevalence of hypertension in pediatric ADPKD was reported as approximately 20–35% in cohort studies conducted two to three decades ago^13–16^, whereas a meta-analysis conducted in 2016 reported a prevalence of 20%^17^. In our cohort, we observed a prevalence of approximately 2.4% among individuals who already had hypertension before or at the time of ADPKD diagnosis. However, it is important to note that the actual prevalence might be higher, as 4.8% of the cohort were already using anti-hypertensive medications prior to the diagnosis of ADPKD. This discrepancy could be attributed to the lack of ICD code data documentation at the time of prescribing medications, as well as the use of anti-hypertensive agents for renal protection by patients with proteinuria.

In children newly diagnosed with ADPKD, the observed incidence of hypertension was approximately 0.7 per 100 person-years. Hypertension in ADPKD is a progressive condition caused primarily by the growth of cysts in the kidneys, which disrupt the normal kidney architecture and compress renal blood vessels and tubules^14,16,18^. As the size and number of renal cysts increase, local ischemia can occur, leading to renin–angiotensin–aldosterone system (RAAS) activation, which in turn results in an increased production of vasoconstrictors and further contributes to elevated blood pressure^18,19^. Furthermore, the prevalence of hypertension in ADPKD owing to the progressive growth of kidney cysts tends to increase over time, regardless of normal or stable kidney function and proteinuria^20^. There is clear evidence of the benefits of controlling hypertension in children, especially those with chronic kidney disease^21^, as well as evidence supporting the efficacy and safety of ACEis and ARBs in pediatric patients, particularly in the context of renal hypertension. Moreover, Wright et al. have demonstrated their superiority over other anti-hypertensive drug classes, particularly in patients with proteinuria^22^. In our cohort, we observed that ACEis or ARBs were the most commonly prescribed anti-hypertensive medications, accounting for 55% of cases. When considering the addition of aldosterone blockers to the treatment regimen, medications targeting the RAAS blockade constituted approximately 63% of the prescribed medications for hypertension management. This suggests that RAAS inhibitors, including ACEis and ARBs, were the preferred choice for blood pressure control in our cohort.

The second most commonly prescribed anti-hypertensive medication category in our cohort was beta blockers, accounting for 22% of cases. Diuretics and CCBs accounted for 6% and 5% of the prescribed medications, respectively. These findings indicate that beta blockers were the next most frequently used anti-hypertensive agents, whereas diuretics and CCBs were less commonly employed for blood pressure management in our cohort of children with ADPKD. In adults with ADPKD, the evidence regarding the comparative efficacy of RAAS inhibitors versus beta-blockers or CCBs for blood pressure control is less conclusive^23,24^. Dual RAAS inhibitor therapy does not offer additional benefits in terms of blood pressure control compared with the use of an ACEi or ARB alone in adults with ADPKD^25^. Diuretics should be used cautiously in ADPKD, as they appear to have detrimental effects on the estimated glomerular filtration rate (eGFR) than when compared to ACEis^26^. Direct comparisons of anti-hypertensive medications in pediatric patients with ADPKD are limited. However, based on studies conducted in adult patients, RAAS inhibitors can be considered a reasonable choice for blood pressure control and slowing the decline in renal function compared with other anti-hypertensive agents. Further research is required to better understand the specific effects of optimal treatment approaches for hypertension in pediatric patients with ADPKD.

ADPKD is primarily caused by pathogenic mutations in the PKD1 or PKD2 genes, which encode for PC-1 and PC-2, respectively. These proteins play crucial roles in cardiac development and function, as evidenced in animal model studies. Moreover, there is an association between ADPKD and congenital heart defects with an overall prevalence of 1.84% reported in a large ADPKD cohort^27^. Notably, mitral valve prolapse has been reported in approximately 26% of patients with ADPKD due to PKD1 mutations^28^. In our cohort, approximately 2.5% of patients had congenital cardiac defects and 0.45% had mitral valve prolapse before or at ADPKD diagnosis. Over a follow-up period of at least one year, the incidence rate was 0.3 per 100 person-year for congenital cardiac defects and 0.2 per 100 person-year for mitral valve prolapse. These rates were higher than those observed in non-ADPKD patients, although the differences were not statistically significant. However, the HR for newly diagnosed congenital cardiac defects was elevated in children with ADPKD. These findings emphasize the importance of routine screening for congenital cardiac defects in patients newly diagnosed with ADPKD. Based on our observations, patients with ADPKD are at an increased risk of congenital cardiac defects; therefore, incorporating routine screening for congenital heart diseases (CHDs) as part of the diagnostic workup for ADPKD is necessary to ensure the early detection and appropriate management of these cardiac complications.

Previous cohort studies revealed an increased incidence of renal and cyst infections (by up to 15–25%) among ADPKD-diagnosed children^5^. In this study, UTI was the most prevalent urinary system diseases (10.7%), and the aHR of newly diagnosed UTI was 3.50 in the pediatric ADPKD population compared to that in the non-ADPKD population. Hematuria, as spontaneous, nephrolithiasis-related or after an abdominal trauma, occurs in 42% of patients with ADPKD, and approximately 10–14% of patients have microscopic or gross hematuria before the age of 16 years^3^. In a previous epidemiological study, isolated hematuria rates in Taiwanese elementary and junior high-school children were 0.13% and 0.18%, respectively^29^. Children with ADPKD also have a higher incidence of nephrolithiasis than that in the general population, which is attributable to anatomical and metabolic abnormalities. In our study, hematuria and nephrolithiasis were markedly more prevalent (aHRs, 5.55 and 13.17, respectively) among children with ADPKD than those without ADPKD, emphasizing the need for routine monitoring of these complications in pediatric patients with ADPKD.

Proteinuria, an indicator of CKD, causes glomerular hypertrophy and worsens tubulointerstitial damage and fibrosis^5^. Notably, a systematic review found a proteinuria prevalence of 20% in children with ADPKD^17^, whereas our cohort exhibited a lower prevalence, and only 2.2% of patients presented with proteinuria prior to ADPKD diagnosis. However, the IRR and aHR for developing proteinuria were 13.9 and 13.5, respectively. Cumulatively, pediatric patients with ADPKD had a significantly higher proteinuria incidence than non-ADPKD patients during follow-up. The cumulative incidence of proteinuria significantly surpassed those in both pediatric ADPKD and non-ADPKD patients over the follow-up period. Overt proteinuria correlates with advanced kidney structural issues and escalates with age in patients with ADPKD. Owing to its therapeutic and prognostic importance, proteinuria monitoring should be the standard in pediatric ADPKD care^5^. We found that 1.2% of children newly diagnosed with ADPKD already had CKD; furthermore, during the course of follow-up, 23 CKD events were documented among the 613 children with ADPKD, signifying a notably higher occurrence compared with that in the non-ADPKD group, which had no CKD events during the same period. A previous meta-analysis^30^ conducted in adults demonstrated that the use of ACEis or ARBs to reduce proteinuria significantly enhanced renal survival in patients with CKD. Although randomized controlled trials in the pediatric population are lacking, it is prudent to consider ACEis or ARBs as primary treatment options for children with ADPKD presenting with proteinuria.

In a previous study, colonic diverticular disease was found to be associated with ADPKD^31^. In this study, there was nearly fourfold higher risk of developing diverticular disease in the intestine or colon after ADPKD was diagnosed. Statins (HMG-CoA reductase inhibitors) not only lower cholesterol, but also enhance renal blood flow and attenuate vascular inflammation through vascular and glomerular nitric oxide production^32^. In our cohort, statin use was approximately tenfold higher among children with ADPKD compared to that in the control group. The incidence of dyslipidemia was also 2.9-fold higher in the ADPKD group, which indicated that statins are used not only used for cholesterol control but also for renal protection in Taiwan. Interestingly, the incidence of hyperuricemia was notably higher in the pediatric ADPKD cohort than that in the non-ADPKD group. A previous study indicated that while hyperuricemia was not an independent factor for renal progression in adult ADPKD, it correlated with reduced eGFR, suggesting that correcting hyperuricemia might slow renal function decline in some patients with ADPKD^33^. The role of hyperuricemia in ADPKD progression remains unclear, necessitating further studies to validate the impact of hyperuricemia control in the ADPKD population.

Approximately 3% of children with ADPKD-causing mutations show very-early onset (VEO) or rapid disease progression^5^. Previous studies have described VEO ADPKD as having a higher risk of developing hypertension and a decline in glomerular filtration rates^34,35^. In our subgroup analysis of ADPKD patients younger than 1.5 years, the incidence rate of hypertension was 0.39 per 100 person-years, compared to none in the non-ADPKD group. ADPKD patients also had significant complications in hematuria (IRR: 5.18, *P* = 0.006) and urinary tract or cyst infections (IRR: 3.12, *P* < 0.001), suggesting the need for careful monitoring these younger patients.

Our large-scale, nationwide, population-based cohort study had several strengths and limitations. Our study used a large population to provide sufficient sample size and statistical power to evaluate the clinical characteristics of ADPKD in the pediatric population. Additionally, it used ICD-9 and ICD-10 codes for case selection, which avoided the need for questionnaires, minimized selection bias via multi-institutional follow-up linkage, and enabled the long-term follow-up of records. However, this study also has some limitations. First, ADPKD often remains asymptomatic in childhood. These patients come to medical attention due to presence of clinical symptoms such as hematuria, proteinuria and hypertension, or diagnosis inferred from family history. However, detailed clinical, disease severity, laboratory, radiographic data and family history were not available from the NHIRD. In addition, the accuracy of the medical codes may have affected the validity of the data. To minimize diagnostic miscoding errors, we only recruited patients with at least one inpatient claim or two outpatient claims for ADPKD and related complications at least 30 days after the index date of ADPKD diagnosis^36^. However, the true incidence and complication rates of ADPKD may have been underestimated because of our strict inclusion criteria or patients not seeking medical care. Future studies might explore the incorporation of additional diagnostic criteria, such as family history or genetic screening results, to provide a more comprehensive assessment of ADPKD incidence. Second, data on personal habits, such as lifestyle habits, smoking, and alcohol use, are not available in the NHIRD. These factors are important and can influence the propensity for cardiovascular and other complications. Finally, the impact of medications such as vasopressin antagonists (tolvaptan) on patients with ADPKD was not analyzed in the present study.

In conclusion, our study provides valuable information on the epidemiology of pediatric ADPKD in Taiwan, including data regarding its national incidence, typical medication use, and associated complications. These findings could be crucial for policymakers in developing clinical guidelines for better ADPKD management. Further prospective studies are also warranted to assess long-term outcomes in our ADPKD population.

### Supplementary Information

Supplementary Information.

## Data Availability

All data generated or analyzed during this study are included in this published article.
