# Supplementary material for: Overview of clinical status, treatment, and long-term outcomes of pediatric autosomal-dominant polycystic kidney disease: a nationwide survey in Taiwan
Source: Sci Rep. 2024 Jul 15;14:16280. doi: 10.1038/s41598-024-67250-z (PMC11251175; doi:10.1038/s41598-024-67250-z)
Supplement: Supplementary file 1 — Supplementary Information. [file 41598_2024_67250_MOESM1_ESM.docx]

Supplement

ETable1. The incidence rate of diseases among patients over 1.5 years old in the ADPKD study population and the non-ADPKD population.

| Outcome | | ADPKD | | | Non ADPKD | |  | IRR | P value |
| --- | --- | --- | --- | --- | --- | --- | --- | --- | --- |
|  |  | n | | IR | n | IR |  |  |  |
| **CV system** | |  |  |  |  |  |  |  |  |
|  | Hypertension | 476 | | 0.80 | 1904 | 0.05 |  | 15.73 | **<0.001** |
|  | Congenital cardiac defects | 481 | | 0.20 | 1924 | 0.11 |  | 1.87 | 0.1993 |
|  | Mitral valve prolapse | 488 | | 0.20 | 1952 | 0.11 |  | 1.86 | 0.2002 |
| **Renal system** | |  |  |  |  |  |  |  |  |
|  | Proteinuria/Albuminuria | 479 | | 0.85 | 1916 | 0.07 |  | 12.87 | **<0.001** |
|  | Hematuria | 464 | | 1.01 | 1856 | 0.17 |  | 6.09 | **<0.001** |
|  | Urinary tract or cyst infections | 447 | | 1.99 | 1788 | 0.59 |  | 3.36 | **<0.001** |
|  | Nephrolithiasis | 488 | | 0.50 | 1952 | 0.02 |  | 20.44 | **<0.001** |
|  | Chronic kidney disease | 482 | | 0.65 | 1928 | 0 |  | - | - |
| **Gastrointestinal system** | |  | |  |  |  |  |  |  |
|  | Diverticulosis | 488 | | 0.13 | 1952 | 0.04 |  | 3.24 | 0.0634 |
|  | Inguinal hernias | 488 | | 0.07 | 1952 | 0.03 |  | 2.02 | 0.4073 |
| **OTHER** | |  |  |  |  |  |  |  |  |
|  | Dyslipidemia | 485 | | 0.51 | 1940 | 0.17 |  | 2.93 | **<0.001** |
|  | Hyperuricemia | 489 | | 0.23 | 1956 | 0.02 |  | 9.47 | **<0.001** |
| **Drug use** | |  |  |  |  |  |  |  |  |
|  | Antihypertensive drug | 462 | | 2.47 | 1848 | 0.35 |  | 7.11 | **<0.001** |
|  | Statins | 488 | | 0.27 | 1952 | 0.02 |  | 10.85 | **<0.001** |
